# Circulating DNA genome-wide fragmentation in early detection and disease monitoring of hepatocellular carcinoma

**DOI:** 10.1016/j.isci.2024.109701

**Published:** 2024-04-09

**Authors:** Shifeng Lian, Chenyu Lu, Fugui Li, Xia Yu, Limei Ai, Biaohua Wu, Xueyi Gong, Wenjing Zhou, Yulong Xie, Yun Du, Wen Quan, Panpan Wang, Li Deng, Xuejun Liang, Jiyun Zhan, Yong Yuan, Fang Fang, Zhiwei Liu, Mingfang Ji, Zongli Zheng

**Affiliations:** 1 Ming Wai Lau Centre for Reparative Medicine, Karolinska Institutet, Sha Tin, Hong Kong SAR of the People’s Republic of China; 2 Unit of Integrative Epidemiology, Institute of Environmental Medicine, Karolinska Institutet, Stockholm, Sweden; 3 Department of Medical Epidemiology and Biostatistics, Karolinska Institutet, Stockholm, Sweden; 4 Cancer Research Institute of Zhongshan City, Zhongshan City People’s Hospital, Zhongshan, People’s Republic of China; 5 Department of Biomedical Sciences and Tung Biomedical Sciences Centre, City University of Hong Kong, Kowloon, Hong Kong SAR of the People’s Republic of China; 6 Department of Precision Diagnostic and Therapeutic Technology, City University of Hong Kong Shenzhen Research Institute, Shenzhen, People’s Republic of China; 7 Department of General Surgery, Zhongshan City People’s Hospital, Zhongshan, People’s Republic of China; 8 Xiaolan Public Health Service Center, Zhongshan, People’s Republic of China; 9 Division of Cancer Epidemiology and Genetics, National Cancer Institute, Rockville, MD, USA

**Keywords:** Clinical genetics, Cancer

## Abstract

Genome-wide circulating cell-free DNA (ccfDNA) fragmentation for cancer detection has been rarely evaluated using blood samples collected before cancer diagnosis. To evaluate ccfDNA fragmentation for detecting early hepatocellular carcinoma (HCC), we first modeled and tested using hospitalized HCC patients and then evaluated in a population-based study. A total of 427 samples were analyzed, including 270 samples collected prior to HCC diagnosis from a population-based study. Our model distinguished hospital HCC patients from controls excellently (area under curve 0.999). A high ccfDNA fragmentation score was highly associated with an advanced tumor stage and a shorter survival. In evaluation, the model showed increasing sensitivities in detecting HCC using ‘pre-samples’ collected ≥4 years (8.3%), 3–4 years (20.0%), 2–3 years (31.0%), 1–2 years (35.0%), and 0–1 year (36.4%) before diagnosis. These findings suggested ccfDNA fragmentation is sensitive in clinical HCC detection and might be helpful in screening early HCC.

## Highlights

• Circulating DNA fragmentation is highly correlated with HCC stage and prognosis
• Approaching diagnosis, cfDNA fragmentation detection sensitivity for HCC increases

## Graphical abstract

**Figure:**
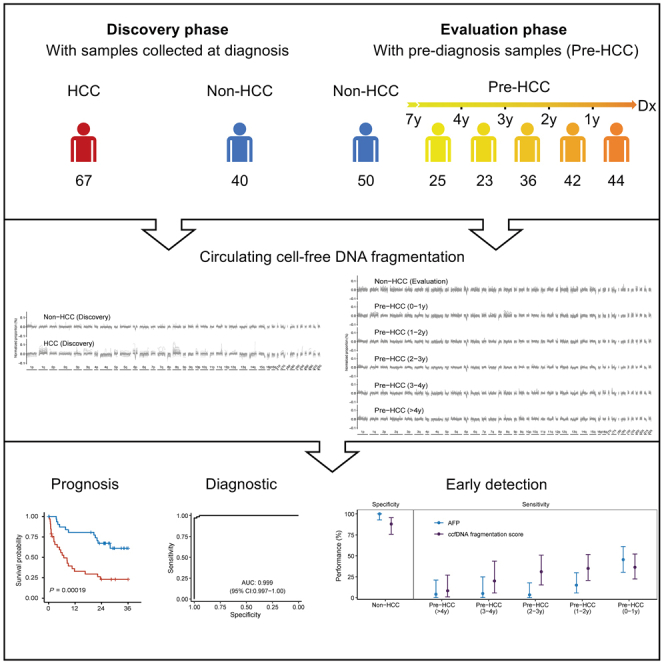

## Introduction

Hepatocellular carcinoma (HCC) is often diagnosed at late stage and has poor survival as no effective therapy is currently available for advanced stage cancer.^1^ Early detection of HCC is important, as patients with early-stage HCC might be treated surgically and have the possibility of being cured.^1^ Among the 905,677 cases of liver cancer diagnosed in 2020 worldwide,^2^ more than 50% are estimated to be attributable to hepatitis B virus (HBV) infection.^3^ Current international guidelines recommend therefore that individuals with chronic HBV infection (i.e., high-risk population) should be screened for HCC by hepatic ultrasound, regardless of serum alpha-fetoprotein (AFP) level, to facilitate early detection.^4^^,^^5^ However, hepatic ultrasound is often unsuccessful to detect early-stage HCC, due to its suboptimal sensitivity and specificity.^6^^,^^7^

The usefulness of circulating cell-free DNA (ccfDNA) shed from solid tumors (a.k.a. liquid biopsy) via analyzing its features, such as somatic mutation^8^^,^^9^ and methylation,^10^^,^^11^^,^^12^ to detect cancer has been studied repeatedly. However, using ‘liquid biopsy’ collected at a time before clinical cancer diagnosis for cancer early detection has been relatively less studied so far, likely due to the challenges related to the ultra-low concentration of these biomarkers at the pre-clinical phase of cancer and the need for large-scale studies with long follow-up time to accumulate just a handful of incident cancer cases.

Studies on ccfDNA fragment size have demonstrated a nucleosome-associated pattern with a peak around 167 bp.^13^ Cancer patients have been shown with increased amount of short ccfDNA fragments, compared to cancer-free controls.^14^^,^^15^ For instance, an enrichment in fragments at the size of 90–150 bp has been shown in cancer.^16^ The analysis of ccfDNA fragmentation profile, through a genome-wide analysis of DNA fragments, has been suggested to be useful for cancer detection.^17^^,^^18^ This approach has for example been reported to have a high sensitivity and specificity in separating clinical HCC cases from controls.^19^^,^^20^ However, the biomarkers having good performance in detecting clinical stage cancer has rarely been evaluated using ‘pre-samples’ in a screening setting where the aim is to identify pre-clinical cancer among a population of high-risk individuals. The potential use of ccfDNA fragmentation as a screening tool for HCC in high-risk populations needs therefore to be evaluated. Finally, as the double-strand DNA sequencing library used in previous studies does not recover single-strand ccfDNA fragments,^21^ whereas cancer ccfDNA can be in both double- and single-stranded.^22^^,^^23^ Thus, here we use a method able to capture double- and single-strand fragments, to unbiasedly evaluate the performance of ccfDNA fragmentation in early cancer detection as well as in prediction of disease prognosis in a high-risk population of HCC.

## Results

### Study participants

The distributions of age and sex are comparable between HCC cases and controls in both the initial model building discovery phase and later in the evaluation phase, since we sampled controls by frequency matching (Table S1). The distributions of Barcelona clinic liver cancer (BCLC) stage were also similar between the HCC cases of the two phases. In the evaluation phase, there were 270 pre-diagnosis (‘pre-samples’) available from 63 incident HCC cases during follow-up, including 25, 23, 36, 42, and 44 cases with at least one pre-sample collected >4 years, 3–4 years, 2–3 years, 1–2 years, or within 1 year before diagnosis, respectively. Among the total 427 blood samples of the cases and controls in the two phases, 24 samples were excluded due to less than 5 million sequencing reads (all were cases in the evaluation phase), resulting in 403 samples in the final analyses.

### ccfDNA fragmentation modeling in the discovery phase

The proportion of ccfDNA fragments with a size of 100–167 nt was higher among clinical HCC cases than controls (Figure S1). This observation is consistent with earlier studies,^15^^,^^16^^,^^21^ suggesting an enrichment of relatively short (100–167 nt) ccfDNA fragments in cancer. We therefore analyzed ccfDNA fragmentation by using the proportion of 100-167 nt ccfDNA in all 504 bins across the genome.

**Figure 1:**
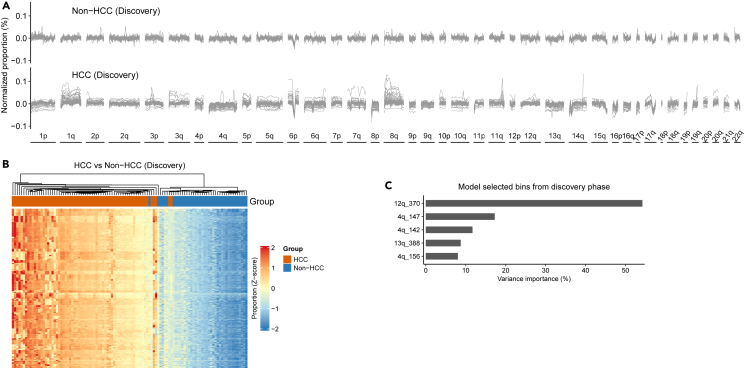
Circulating cell-free DNA fragmentation modeling in the discovery phase (A) The proportion of ccfDNA fragment with a size of 100–167 nt in all 504 bins across the genome at a bin size of 5-Mb. X axis was the 504 bins across the genome along chromosome arms. Y axis was the normalized fragmentation proportion calculated by subtract each bins proportion with the mean proportion of each sample. (B) Clustering result using 504 bins ccfDNA (100-167 nt) proportion in the discovery phase. (C) The ccfDNA (100-167 nt) proportion of five bins that survived the LASSO modeling for distinguishing HCC cases and non-HCC controls in the discovery phase, forming the ccfDNA fragmentation model, and the relative importance of each bin on distinguishing HCC cases from controls. Y axis denotes the five bins, named using chromosome arm and bin number of each bin.

In contrast to a relatively uniform profile in controls, we observed a highly varying ccfDNA fragmentation pattern in clinical HCC cases across the genome, most obviously on 1q and 8q (Figure 1A). The ratio of the proportion among cases to that of controls ranges between 1.50 and 2.38 across the 504 bins (Figure S2). Hierarchical clustering analysis showed an excellent separation between HCC cases and controls using collectively the fragmentation features across the 504 bins (Figure 1B). Next, we used LASSO to build a ccfDNA fragmentation model for HCC detection. The resulting model included 100-167 nt ccfDNA proportions from five bins, namely 12q_370, 4q_147, 4q_142, 13q_388, and 4q_156. The proportional contributions of these bins to the model were 54.2%, 17.3%, 11.7%, 8.8%, and 8.0%, respectively (Figure 1C).

**Figure 2:**
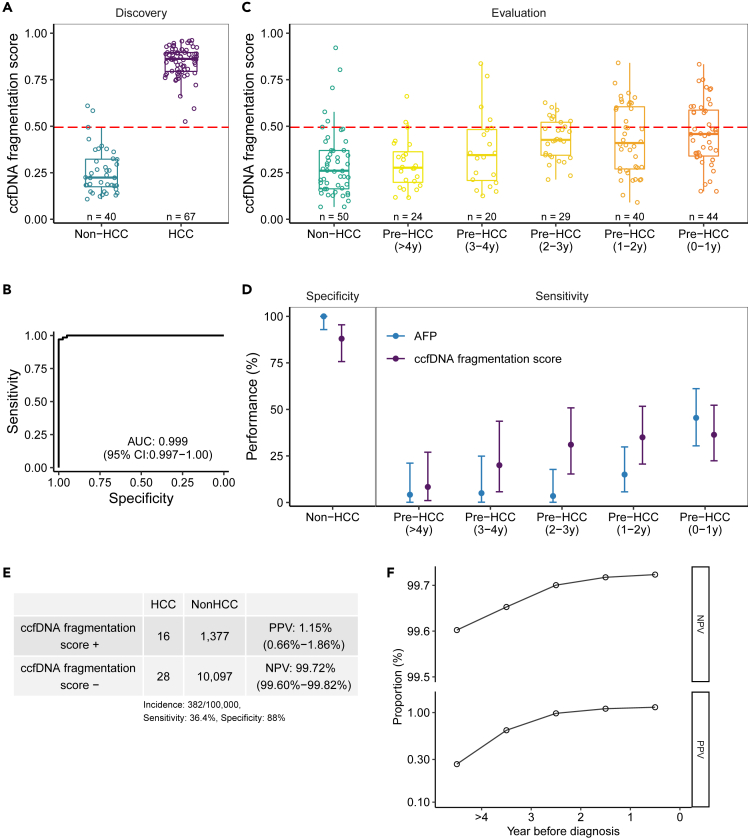
Evaluation of the ccfDNA fragmentation model for early detection of HCC (A) Distributions of the ccfDNA fragmentation model score in HCC cases and controls in the discovery phase. The red dashed line represents the cut-off score of 0.494, which corresponds to two misclassified controls. (B) Area under the receiver operating characteristic curve (AUC) of the ccfDNA fragmentation model for detecting HCC in the discovery phase. (C) Distributions of the model score in pre-HCC cases and controls in the evaluation phase. Pre-HCC samples were classified into 5 intervals at >4, 3–4, 2–3, 1–2, and 0–1 year before diagnosis. For cases with multiple samples in a given time interval, mean of the model scores was used. The red dashed line represents the cut-off score of 0.494 derived from the discovery phase. (D) Specificity, sensitivity and corresponding 95% confidence intervals (CI) in the evaluation phase. Pre-HCC samples were classified into 5 intervals at >4, 3–4, 2–3, 1–2, and 0–1 year before diagnosis. Bars indicate 95% CI. (E) Positive predictive values (PPV) and negative predictive value (NPV) for the ccfDNA fragmentation model calculated in a scenario where the annual incidence of HCC is 382 per 100,000 person-years (corresponding to the HCC incidence rate among both sex HBV-seropositive population in the screening cohort), with specificity of 88% and sensitivity of 36.4%. (F) The PPVs and NPVs of the ccfDNA fragmentation by time to diagnosis among HBV-seropositive population.

In the discovery phase, the mean score (±SD) derived from the fragmentation model were 0.846 (±0.083) and 0.258 (±0.124) among HCC cases and controls, respectively. At a cutoff score of 0.494, cases were separated excellently from controls, with an area under curve (AUC) of 0.999 and 95% confidence interval (CI) of 0.997–1.000 and a specificity of 95% (two controls misclassified as cases) (Figures 2A and 2B).

### Evaluation of the ccfDNA fragmentation model in HCC early detection

In the evaluation of the performance of the ccfDNA fragmentation model in detecting early HCC, we found increasing scores in the ‘pre-samples’ of the HCC cases by closer time to diagnosis, with mean scores of 0.299, 0.373, 0.432, 0.431, and 0.461 for samples collected >4 years, 3–4 years, 2–3 years, 1–2 years, and within 1 year before diagnosis, respectively (Figure 2C). At the cutoff of 0.494, the sensitivities (95% CI) were 8.3% (1.0%–27.0%), 20.0% (5.7%–43.7%), 31.0% (15.3%–50.8%), 35.0% (20.6%–51.7%), and 36.4% (22.4%–52.2%), respectively, during these five time-windows before HCC diagnosis (Figure 2D). Among the controls of the evaluation phase, the mean score was 0.300 and, using the same cutoff, six controls were misclassified as cases leading to a specificity of 88% (75.7%–95.5%) (Figure 2D). For comparison, serum alpha-fetoprotein (AFP), at a cutoff value of 20 ng/mL, had a specificity (95% CI) of 100% (92.9%–100%) and sensitivities (95% CIs) of 4.2% (0.1%–21.1%), 5.0% (0.1%–24.9%), 3.4% (0.1%–17.8%), 15.0% (5.7%–29.8%), and 45.5% (30.4%–61.2%), respectively (Figure 2D).

### Positive predictive value (PPV) and negative predictive value (NPV)

During 2012–2019, we observed 81 incident HCC cases among the 21,189 person-years accumulated during the follow-up of the 2,893 HBV seropositive in the screening, leading to an incidence rate of HCC 382 per 100,000 person-years. Among the 81 cases, 44 had a pre-HCC sample collected <1 year before diagnosis. At a specificity of 88% (observed in the controls of our evaluation phase), a sensitivity of 36.4% (observed in our pre-HCC samples collected <1 year before diagnosis), an HCC incidence rate of 382 per 100,000 person-years, and at a score cutoff of 0.494, our ccfDNA fragmentation model yielded a PPV of 1.15% (95% CI: 0.66%–1.86%) and an NPV of 99.72% (95% CI: 99.60%–99.82%) in identifying HCC cases one year before clinical diagnosis (Figure 2E). As expected, the PPV and NPV with a cutoff of 0.494 increased by closer time of the pre-HCC samples to diagnosis (Figure 2F).

**Figure 3:**
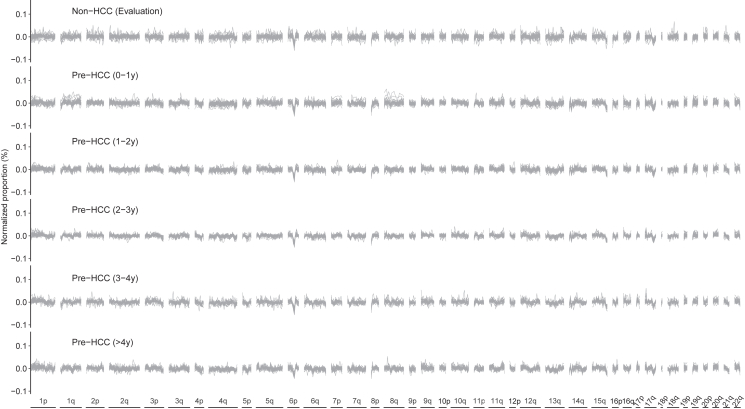
Circulating cell-free DNA fragmentation in the evaluation phase The proportion of ccfDNA fragments with size of 100–167 nt in all 504 bins across the genome at a window of 5-Mb in size in the evaluation phase. X axis is the 504 bins across the genome. Y axis is the normalized proportion calculated by subtract each bins proportion with the mean proportion of each sample.

We then tried to understand potential reasons underlying the relatively low PPV of the ccfDNA fragmentation model in early detection of HCC. We found that only the pre-HCC samples collected within 1 year before cancer diagnosis showed somewhat similar patterns as those of the clinical HCC cases in the discovery phase, such as the variations on 1q and 8q, whereas pre-HCC samples collected earlier than 1 year before diagnosis showed largely similar patterns as the controls (Figure 3). Similarly, the hierarchical clustering analyses using the fragmentation failed to separate pre-HCC samples from samples of the controls (Figure S3).

### CcfDNA fragmentation and HCC prognosis

**Figure 4:**
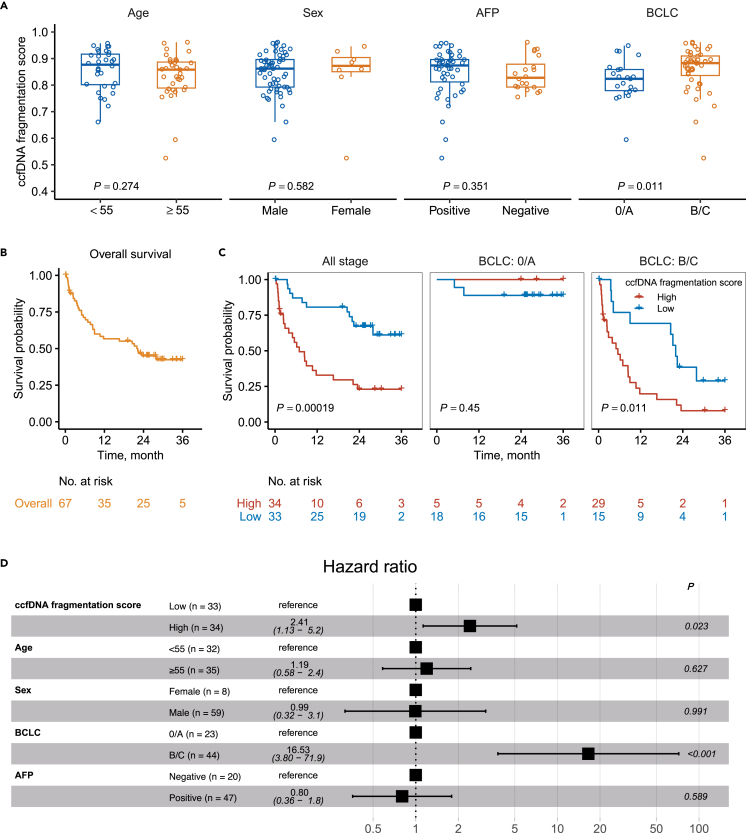
Circulating cell-free DNA fragmentation model and HCC patient survival (A) Distribution of the score by age, sex, AFP, and BCLC stage. (B) Overall survival among 67 HCC patients over 36 months of follow-up. (C) HCC survival by the score (high [≥0.862] vs. low [<0. 0.862], based on the median among all HCC cases) over 36 months of follow-up, and stratified by BCLC stage. (D) Hazard ratios derived from a Cox regression model including the ccfDNA fragmentation model score, age, sex, BCLC stage, and AFP status.

Among the HCC cases in the discovery phase, the ccfDNA fragmentation scores did not differ by age (<55 vs. ≥ 55), sex, or AFP positivity (Figure 4A). However, there was a positive correlation between the score and BCLC stage (stage B/C: 0.860; and stage 0/A: 0.819; *p* = 0.011). During a 36-month follow-up of the 67 cases, we observed 35 deaths (52.2%) and a median survival of 22.2 months (Figure 4B). HCC patients with a score above the median (0.862) (*n* = 34) had a poorer survival than patients with a score below the median (*n* = 33) (*p* < 0.001). A high score was also associated with a poorer survival in advanced disease (stage B/C) in the stratified analysis by BCLC stage (Figure 4C). After adjustments for age, sex, BCLC stage, and AFP, a higher score was associated with a higher risk of death (hazard ratio 2.41; 95% CI: 1.13–5.2; *p* = 0.023; Figure 4D).

## Discussion

Using a machine learning method, we constructed a ccfDNA fragmentation-based model to detect HCC and evaluated this model using pre-HCC samples from a population-based cancer screening program. To the best of our knowledge, this is the first study to evaluate the utility of ccfDNA fragmentation on HCC early detection using pre-diagnosis samples. We found that the model score was associated with HCC stage and may be useful in clinical diagnosis of HCC. The model can also predict survival in advanced HCC patients, which is consistent with a previous fragmentation study on lung cancer.^18^ There was a trend of increasing sensitivity using samples approaching cancer diagnosis, suggesting the potential role of ccfDNA fragmentation to screen HCC in high-risk populations (i.e., with chronic HBV infection).

We observed a greater variation of the ccfDNA fragmentation profile in hospital HCC cases compared with non-cancer controls, especially on 1q and 8q. This result was consistent with a previous study^20^ and suggested that our shallow whole genome sequencing data is able to uncover fragmentation feature of HCC cases. Losses of tumor suppressor genes located on chromosomes 4q, 12q and 13q in early HCC patients^24^ may contribute to the corresponding chromosome bins being included in our ccfDNA fragmentation model. HCC is aggressive and has an estimated tumor volume doubling time of about 4.6 months.^25^ The ccfDNA fragmentation will therefore be expected to change fast in a short time before clinical diagnosis. This explains our observation, at least in part, that only the pre-HCC samples collected within 1 year before cancer diagnosis showed a somewhat similar pattern as that of the diagnosed HCC cases; and that pre-HCC samples collected >1 year before diagnosis showed similar patterns as that of the non-cancer controls.

PPV is a key parameter in evaluating the effectiveness of a screening program. The PPV for ccfDNA fragmentation was reported with a value range from 1.9% to 3.9% using simulated lung cancer screening data.^18^ In our study, the PPV value for ccfDNA fragmentation in HCC screening was estimated to be 1.15% (95% CI: 0.66%–1.86%) using samples collected within 1 year before cancer diagnosis. The PPV estimates in our study were based on a real-world practice of HCC screening in a high-risk HBV-positive population and therefore provides a useful reference to conduct a prospective HCC screening project. The relatively low PPV suggested that the utility of ccfDNA fragmentation in HCC screening in high-risk populations so far may be limited. More studies are needed before taking the ccfDNA fragmentation into a population cancer screening.

In conclusion, ccfDNA fragmentation may be highly associated with tumor burden and could help HCC prognosis prediction. Genome-wide fragmentation profile of ccfDNA may be helpful, when combined with other biomarkers, for early HCC detection in high-risk populations.

### Limitations of the study

One limitation of our study is the relatively small number of pre-HCC cases in the evaluation phase, even after following up on a large and high-risk population for over 7 years. Second, as our study focused on HBV-positive population, the generalizability of our results to other populations of non-HBV-related HCC needs further evaluation.

## STAR★Methods

### Key resources table

**Table:** 

REAGENT or RESOURCE	SOURCE	IDENTIFIER
**Biological samples**		

Human plasma samples	Zhongshan City People’s Hospital	See method details

**Critical commercial assays**		

QIAamp® MinElute® ccfDNA Mini Kit	QIAGEN	Cat# 55284

**Deposited data**		

Raw data	This paper	GSA-human: HRA006769

**Software and algorithms**		

bcl2fastq2	Illumina	V2.20.0
BBDuk	Bushnell, Brian. 2014^26^	https://www.osti.gov/biblio/1241166
Burrows-Wheeler Aligner (BWA)	Li and Durbin, 2009^27^	http://bio-bwa.sourceforge.net/
R, v4.1.2	The R Foundation	https://www.r-project.org/

### Resource availability

#### Lead contact

Further information and requests for resources and reagents should be directed to and will be fulfilled by the Lead Contact, Zongli Zheng (zongli.zheng@cityu.edu.hk).

#### Materials availability

This study did not generate new unique reagents.

#### Data and code availability

• The raw sequence data reported in this paper has been deposited in the Genome Sequence Archive in National Genomics Data Center, China National Center for Bioinformation/Beijing Institute of Genomics, Chinese Academy of Sciences (GSA-Human: HRA006769) that are publicly accessible at https://ngdc.cncb.ac.cn/gsa-human.^28^^,^^29^
• This paper does not report original code.
• Any additional information required to reanalyze the data reported in this paper is available from the lead contact upon request.

### Experimental model and study participant details

#### Study participants

This study included a discovery phase and an evaluation phase. In the discovery phase, we explored the potential use of ccfDNA fragmentation analysis in differentiating clinical HCC patients from controls in a hospital-based case-control study, including 67 HCC cases and 40 controls. The controls were randomly selected from a prospective cohort of 2,893 HBV-seropositive individuals participating in the liver cancer screening trial in Zhongshan, China (NCT02501980, ClinicalTrials.gov) between 2012 and 2019.^30^ The cases and controls were both tested positive for serum HBV surface antigen (HBsAg) and frequency-matched by age and sex. In the evaluation phase, we assessed the use of ccfDNA fragmentation analysis in differentiating pre-clinical HCC from controls in a nested case-control study based on the same cohort, including 63 incident HCC cases with available pre-diagnosis blood samples and 50 controls frequency-matched to the cases on age at recruitment to the screening trial (±1 year), sex, and time from last blood collection to diagnosis or end of follow-up (±3 months). In the discovery phase, there were 59 males and 8 females among HCC cases, and 36 males and 4 females among controls, with mean ages of 55.2 and 55.1 years, respectively. In the evaluation phase, there were 58 males and 5 females among pre-clinical HCC cases, and 46 males and 4 females among controls, with mean ages of 55.7 years for both groups. The non-HCC controls in both the discovery and evaluation phases were HBV-seropositive individuals who did not have a diagnosis of HCC until the end of the follow-up. All samples were obtained under approved protocols with informed consent from all participants for research use (ZSKY2012 [02], Zhongshan People’s Hospital). The study was also approved by the Swedish Ethical Review Authority (DNR: 2020–02803).

### Method details

#### Sample processing and DNA sequencing

Venous peripheral blood samples were collected using the K2-EDTA tube. The plasma samples from the population screening cohort were collected by centrifuging at 1,600 × g at room temperature for 10 min. The plasma samples from hospital HCC samples were collected before any treatment and separated from the buffy coat using centrifugation at 1,600 × g at room temperature for 10 min, followed by a second centrifugation at 16,000 × g at 4°C for 10 min to remove remaining cellular debris. All plasma samples were stored at either −20°C or −80°C for future analysis. We extracted ccfDNA from ∼1 mL plasma using the QIAamp MinElute ccfDNA Mini Kit (Cat. No. 55284, QIAGEN, Germantown, MD). We constructed the sequencing library using a new method that can convert both single-stranded and double-stranded DNA fragments into a sequencing library. Briefly, extracted DNA was first de-phosphorylated using FastAP (Thermo Fisher Scientific, MA, USA) and incubated at 37°C for 15 min, 75°C for 10 min, and 95°C for 3 min, and immediately cooled down on ice water. Next, the product was ligated with a unique molecule index (UMI)-containing an adaptor that can ligate the 3′ end of single-strand DNA. The reaction was then cleaned up with 1.5 x Agencourt AMPure XP beads (Beckman Coulter, CA, USA). The purified product was then phosphorylated by T4 Polynucleotide Kinase with ATP and incubated at 37°C for 30 min, 65°C for 20 min, 95°C for 3 min, and immediately cooled on ice water, followed by ligation with another UMI-adaptor that can ligate the 5′ end of single-strand DNA. Finally, the product was amplified by 10 cycles of PCR using sequencing platform (Illumina) adaptor primers with sample barcodes and purified by 1.0 x Agencourt AMPure XP beads. The library was quantified by real-time PCR with the KAPA Library Quantification Kits for Illumina System and sequenced on the NovaSeq 6000 System (Illumina, Inc., San Diego, CA, USA).

We de-multiplexed the raw FASTQ data using bcl2fastq2, trimmed adaptors using BBDuk,^26^ and extracted the UMIs using in-house scripts. We aligned the cleaned FASTQ sequences to human reference genome (hg38) using BWA MEM.^27^ BAM files were then lifted over to hg19 using the liftOver function of the rtracklayer R package. Read pairs with MAPQ score below 30 were excluded from downstream analyses.

### Quantification and statistical analysis

#### Data analysis

A total of 26,236 non-overlapping 100-Kb bins located on the 39 non-acrocentric arms of the hg19 autosomes were adopted, according to a previous study.^17^ These bins are associated with open (A) and closed (B) compartments demonstrated in the high-throughput sequencing chromosome conformation capture (Hi-C) data.^31^ The regions of low mappability and Duke blacklisted were removed. We then combined bins approximate to each other and ended up with a total of 504 bins at a window of 5-Mb in size. These bins were labeled by chromosome arm and a sequential bin number, namely from 1q_1 to 22q_504. To adjust for the fluctuation of sequencing coverage in our low-pass whole genome sequencing, we calculated ccfDNA fragmentation across the 504 bins by dividing the number of fragments with a size of 100–167 nucleotides by the total number of ccfDNA fragments of all sizes within a bin. We did not correct for the GC content considering the low GC bias in shallow whole-genome sequencing data, and that the UMI used for de-multiplexed could further reduce the GC bias.

We first analyzed the use of the ccfDNA fragmentation in differentiating clinical HCC cases from HCC-free HBV-seropositive controls in the discovery phase. The Least Absolute Shrinkage and Selection Operator (LASSO)^32^ based machine learning approach was used for training the model. 5-fold cross-validation by resampling was used to determine the optimal value of lambda (λ) penalty using the caret R package. We then evaluated the ccfDNA fragmentation model in the evaluation phase using serial blood samples collected before HCC diagnosis (i.e., pre-HCC samples). For cases and controls with multiple samples in a specific time window, the mean value of the model score was used. In both phases, we used area under curve (AUC), positive predictive value (PPV), and negative predictive value (NPV), in addition to specificity and sensitivity, to evaluate the diagnostic performance of the ccfDNA fragmentation model. Finally, among HCC cases of the discovery phase, we compared the model scores by age, sex, AFP, and Barcelona clinic liver cancer (BCLC) stage using Wilcoxon rank-sum test. We also assessed the association of ccfDNA fragmentation with overall survival after cancer diagnosis using Kaplan-Meier method and Cox model. Overall survival time was calculated from the date of diagnosis until the date of death or end of follow-up over 36 months, whichever occurred first.

Statistical analyses were conducted using R version 4.1.2, including R packages caret, pROC, ComplexHeatmap, survival, and epiR. All *p* values are two-sided and a *p* value of less than 0.05 was considered statistically significant.
