# Supplementary material for: Circulating DNA genome-wide fragmentation in early detection and disease monitoring of hepatocellular carcinoma
Source: iScience. 2024 Apr 9;27(5):109701. doi: 10.1016/j.isci.2024.109701 (PMC11053305; doi:10.1016/j.isci.2024.109701)
Supplement: Document S1. Figures S1–S3 and Table S1 [file mmc1.pdf]

iScience, Volume *27*

Supplemental information

Circulating DNA genome-wide fragmentation in early detection and disease monitoring of hepatocellular carcinoma

Shifeng Lian, Chenyu Lu, Fugui Li, Xia Yu, Limei Ai, Biaohua Wu, Xueyi Gong, Wenjing Zhou, Yulong Xie, Yun Du, Wen Quan, Panpan Wang, Li Deng, Xuejun Liang, Jiyun Zhan, Yong Yuan, Fang Fang, Zhiwei Liu, Mingfang Ji, and Zongli Zheng

Table S1. Baseline characteristics of participants in the discovery and evaluation phases, related to STAR Methods.

Characteristics	Discovery phase			Evaluation phase		
	HCC patients (n = 67)	Non-HCC (n = 40)	*P*	Pre-HCC patients (n = 63)	Non-HCC (n = 50)	*P*
**Age at baseline**						
Mean (SD)	55.2 (9.43)	55.1 (6.53)	0.926 [a]	55.7 (6.52)	55.7 (7.21)	0.955 [a]
**Sex, n (%)**						
Female	8 (11.9%)	4 (10.0%)	1.00	5 (7.9%)	4 (8.0%)	1.00
Male	59 (88.1%)	36 (90.0%)		58 (92.1%)	46 (92.0%)	
**AFP, n (%)**						
Negative	20 (29.9%)	40 (100%)	< 0.001	41 (65.1%)	50 (100%)	< 0.001
Positive	47 (70.1%)	0 (0%)		22 (34.9%)	0 (0%)	
**BCLC stage, n (%)**						
0/A	23 (34.3%)	-		21 (33.3%)	-	0.081
B	22 (32.8%)	-		19 (30.2%)	-	
C	22 (32.8%)	-		16 (25.4%)	-	
D	0 (0%)	-		5 (7.9%)	-	
Unknown	0 (0%)	-		2 (3.2%)	-	

[a] *P* value was determined by a 2-sided t-test. Other *P* values were determined by a Fisher's Exact test.

Abbreviations: HCC, hepatocellular carcinoma; Pre-HCC, pre HCC diagnosis samples; BCLC stage, Barcelona clinic liver cancer stage

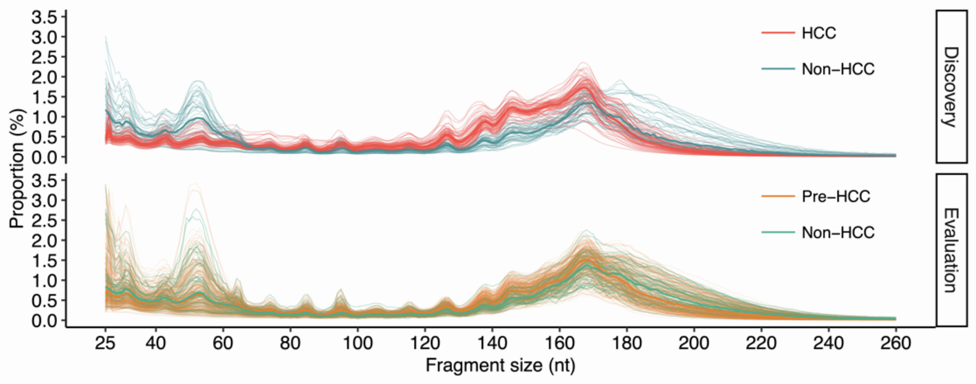

Figure S1. Circulating cell-free (ccfDNA) fragment length, related to Figures 1 and 3. The ccfDNA fragment length distribution by case-control status in the discovery and evaluation phases.

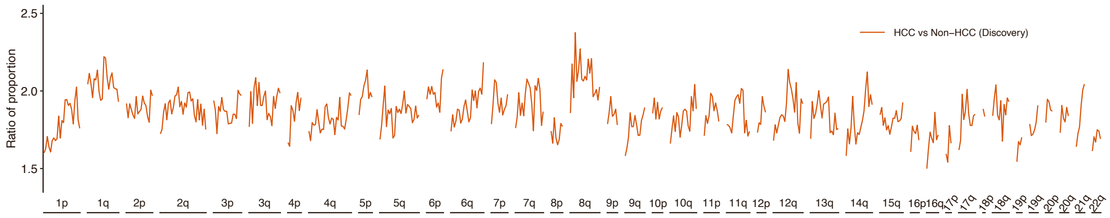

Figure S2. Ratio of fragment proportion by bins in discovery, related to Figure 1.

Ratio of the ccfDNA (100-167nt) proportion comparing hospital HCC cases to controls among 504 bins across the genome in the discovery phase.

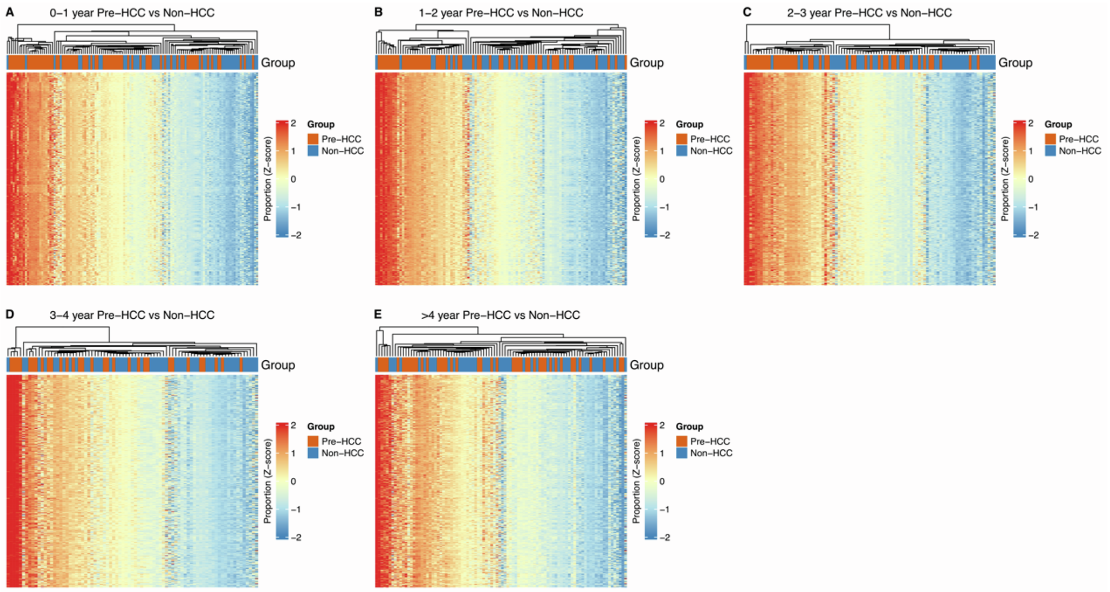

Figure S3. Hierarchical clustering analyses in the evaluation phase, related to Figure 3. Clustering results using ccfDNA fragmentation with pre-HCC samples collected at the interval of 0-1 year (A), 1-2 year (B), 2-3 year (C), 3-4 year (D), and >4 year (E) before diagnosis in the evaluation phase.
